# The Narrative Impact of Active Video Games on Physical Activity Among Children: A Feasibility Study

**DOI:** 10.2196/jmir.6538

**Published:** 2016-10-14

**Authors:** Amy Shirong Lu, Tom Baranowski, S Lee Hong, Richard Buday, Debbe Thompson, Alicia Beltran, Hafza Razak Dadabhoy, Tzu-An Chen

**Affiliations:** ^1^ College of Arts, Media & Design Department of Communication Studies Northeastern University Boston, MA United States; ^2^ Bouvé College of Health Sciences Department of Health Sciences Northeastern University Boston, MA United States; ^3^ USDA/ARS Children’s Nutrition Research Center Department of Pediatrics Baylor College of Medicine Houston, TX United States; ^4^ Information Control Company Columbus, OH United States; ^5^ Archimage Inc Houston, TX United States; ^6^ McGovern Medical School The University of Texas Health Science Center at Houston Houston, TX United States

**Keywords:** video games, narration, behavior, child health, child obesity, motivation

## Abstract

**Background:**

Active video games (AVGs) capable of inducing physical activity offer an innovative approach to combating childhood obesity. Unfortunately, children’s AVG game play decreases quickly, underscoring the need to identify novel methods for player engagement. Narratives have been demonstrated to influence behaviors.

**Objective:**

The objective of this study was to test the hypothesis that a narrative would motivate increased AVG play, though a feasibility study that investigated the motivational effect of adding a previously developed narrative cutscene to an originally nonnarrative AVG, *Nintendo Wii Sports Resort: Swordplay Showdown*.

**Methods:**

A total of 40 overweight and obese 8- to 11-year-olds equally divided by sex played the AVG. Half (n=20) were randomly assigned to a narrative group that watched the narrative cutscene before game play. The other half played the game without watching it.

**Results:**

Children in the narrative group had significantly (*P*<.05) more steps per 10-second period (mean 3.2, SD 0.7) and overall (mean 523, SD 203) during game play compared with the nonnarrative group (10-second period: mean 2.7, SD 0.7; overall: mean 366, SD 172).

**Conclusions:**

The AVG with narrative induced increased physical activity. Additional research is needed to understand the mechanisms through which narrative increases physical activity during AVG game play.

## Introduction

### Background and Theory

Children in the United States spend more time with electronic media than any other activity except sleep [1]. Their daily video game play has tripled over the past decade [1]. Traditional video games are sedentary [2] and the likelihood of getting physical activity from health education games is low [3]. Active video games (AVGs), or “interactive video or electronic games that feature player movement, such as would occur in ‘real-life’ exercise participation” [4], capable of inducing moderate physical activity levels may be a method for increasing youth physical activity [5-7].

A critical issue for AVG interventions, however, is that players typically do not play them for sufficiently long durations [8]. Innovative approaches to promote continued game play are needed to address this problem. A possible solution is the telling of narratives, or stories, one of the most distinctive characteristics of human social groups [9]. Narratives may have a crucial role in motivating increased game play in AVGs through their immersive properties, resulting in increased engagement, but their role has not been systematically investigated [10].

To our knowledge, this is the first feasibility study to systematically investigate the effect of narrative on children’s AVG play, testing the hypothesis that narratives will increase physical activity during AVG play. A professionally developed narrative cutscene was developed (ie, a brief, animated movie clip) for an existing AVG requiring trunk movement at a moderate level of physical activity. Children aged 8-11 years played either the narrative or the nonnarrative version of the game by either watching or not watching the narrative cutscene before game play.

Childhood obesity is a worldwide problem [11], which increases the risk of certain cancers [12-14], shortens life span [15], impedes functional ability [15], diminishes quality of life [15], and tracks into adulthood [16]. Physical activity is critical to preventing childhood obesity [17,18]. US physical activity guidelines recommend 60+ minutes of age-appropriate, enjoyable, mostly moderate or vigorous daily physical activity for children [17]. Yet few children meet these guidelines [18]. Most physical activity interventions have not achieved these effects; lack of access and motivation were identified as key challenges [19-21].

Active video games could provide an innovative method of increasing physical activity with promising health outcomes for many children [22]. As of 2014, an average US household owned at least one dedicated gaming device [23]. All major game console manufacturers offer controllers that can be used as exercise equipment [24]. In 2015, a typical child in the United States aged 8-12 years spent 1.33 hours/day playing video games and 81% of them had a video game console at home [25]. Access to these types of equipment may encourage physical activity among children who live in unsafe neighborhoods that lack accessible outdoor alternatives [4,26,27]. Replacing sedentary activities (eg, entertainment-oriented video games) with AVG play may increase physical activity, thus reducing obesity risk [28].

While AVGs may prevent childhood obesity by increasing physical activity levels, reported AVG play duration varies. One study found that a quarter of young players played AVGs for 2 days a week in bouts of 50 minutes on average [29], whereas another showed that the daily average time spent playing AVGs was only 5 minutes (SD 13.1) for adults and 8 minutes (SD 14.7) for children [30]. Despite game companies’ continued high level of investment in “AAA” games, that is, games with the highest development budgets, quality, and levels of promotion [31], most players did not play one game completely before starting a new one [8]. The obesity-combating potential of AVG cannot be realized if players do not play in sufficient dosage [32-34].

**Figure 1:**
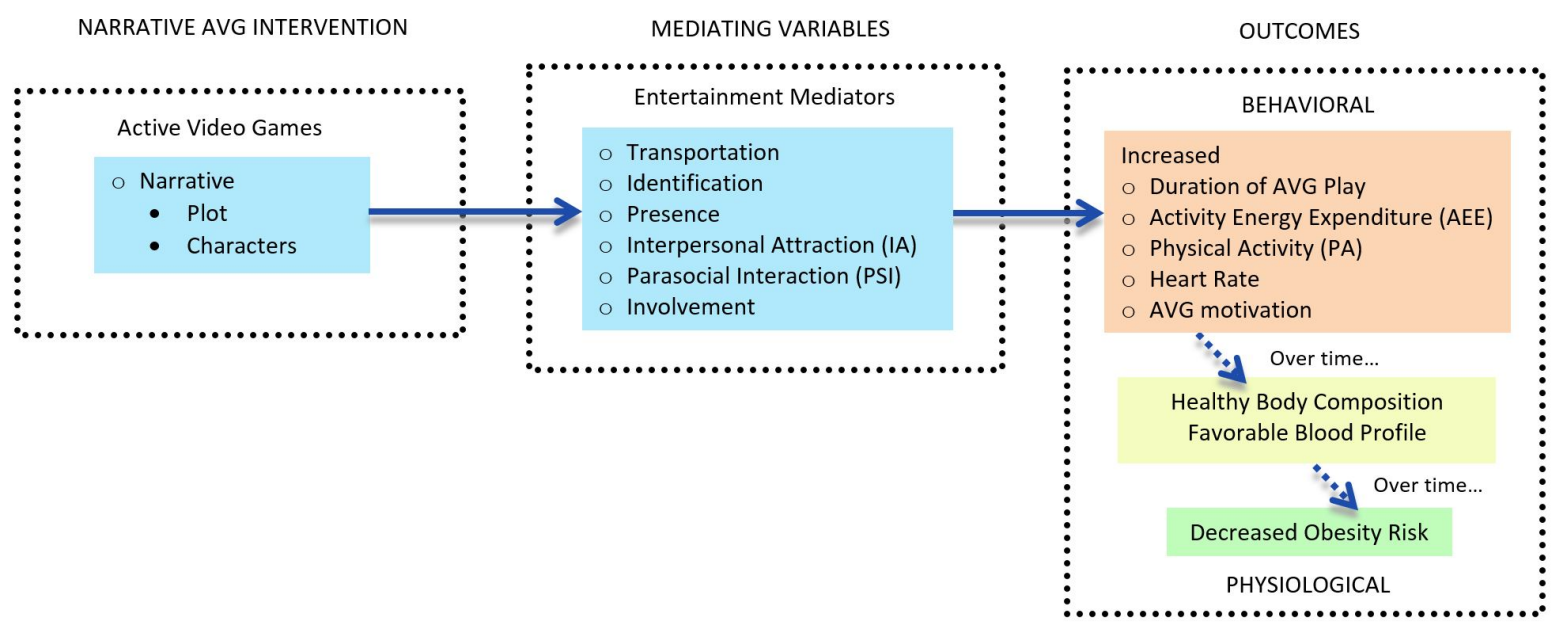
Conceptual model of the mechanisms for narrative effects. AVG: active video game.

Approaches are needed to enhance physical activity resulting from AVG play. Narratives possess unique motivational properties that may encourage AVG play [10]. Although narratives appear in some health video games [35], most were simply used as background context at the beginning of a game and not well adapted throughout the game play. Few AVGs capable of achieving a moderate physical activity level have incorporated narratives [36]. One of the most basic forms of human communication [37], a narrative can be defined as any two or more events arranged in a chronological or causal order [38]. The ability to enjoy narratives is universal [39]. On the psychological level, narratives have a significant impact on cognition, affect, and, potentially, health behavior [40] through transportation, a unique immersive quality that enables suspension of disbelief [41], instills vivid personal experiences [42,43], and helps create deep affection for the characters [44]. The addition of compelling narratives to AVGs could foster strong intrinsic motivation, defined as motivation that comes from inside an individual rather than from outside, to play by reducing cognitive load [45]; engendering arousal and attention [46]; eliciting character identification [47]; and absorbing players in an immersive fictional world [48] that promotes physical activity as necessary and fun [49]. Narratives also encourage players in their role as characters to enhance and maintain their physical activity [50]. On the behavioral level, AVGs with well-constructed stories may elicit desirable behavioral consequences, such as a higher level of physical activity than that elicited by nonnarrative AVGs. Figure 1 illustrates the conceptual model of the potential mechanism for narrative effects. A more detailed explanation of this conceptual model can be found elsewhere [10]. As part of the initial approach in addressing this gap in scientific understanding, this feasibility study tested the hypothesis that a child-friendly narrative would increase physical activity during AVG play.

### Hypothesis

The narrative version of the AVG will result in a higher level of physical activity measured by steps/second count, total steps count, play duration, and total energy expenditure than the nonnarrative version.

## Methods

### Design

This study used a 2-arm randomized controlled design with assessments of children’s AVG play during and after the sessions. Children were randomly assigned to narrative (n=20) or nonnarrative (n=20) groups.

### Sample

Inclusion criteria were as follows: age 8-12 years, between the 85th and 99th percentiles for body mass index (BMI), ability to speak and understand English, and physically able to play the selected AVG. This group was targeted because (1) obese children in this age group are highly likely to become obese young adults [51] and interventions have had effects primarily among the overweight and obese [52,53]; (2) children younger than 8 years have cognitive limitations in responding to questionnaires [54], while children older than 12 years have entered early adolescence and may require different intervention strategies [55]; (3) English is a commonly taught language among children, including migrant children living in the study region; and (4) higher BMI percentile could prevent them from playing the AVG safely. Exclusionary criteria were not speaking or understanding English, having medical or physical problems that prevented AVG game play (eg, epilepsy, using orthopedic devices), or morbid obesity (BMI percentile ≥ 99). Special attention was given to recruiting African American and Hispanic children, the racial and ethnic groups linked to higher rates of obesity [11].

The Institutional Review Boards of Northwestern University and the Baylor College of Medicine approved the research protocols. Children were recruited from mostly lower-income public schools in Chicago and a participant database in Houston. Parents provided written informed consent and children provided written informed assent.

### Intervention: Narrative Development and Format

Prior research has used both quantitative and qualitative methods (surveys and cognitive interviews) to explore child preferences for the type of narrative genres (eg, adventure, fable, mystery, comedy) and story plots. A total of 4 plots were developed to presage the selected AVG, *Swordplay: Showdown* (Nintendo Co, Ltd, Kyoto, Japan), by a professional media production company. *Swordplay: Showdown* requires players to wave a remote game controller as a sword to knock out enemies coming at them in different environments (eg, bridge, mountain, ruins). Because the essential movement was to wield a sword, “sword fighting” became the theme of the 4 narratives. An earlier formative observational study suggested that children playing this game were more likely to engage in trunk movement instead of just moving their arms or wrists.

A total of 20 children were recruited from the Chicago metro area. Of the 4 narrative plots, *The Door* was the children’s preferred story line. *The Door* tells the story of an ordinary child mysteriously absorbed into a strange world full of cartoon stickmen carrying swords. Results of cutscene testing and development are reported elsewhere [56]. To ensure the narrative would appeal to a diverse audience, character race and ethnic background as well as the plot and background cues were made racially and ethnically ambiguous [57-59].

The selected plot was fully developed and entitled *The Door*. *The Door* included information about the health benefits of physical activity, delivered in a narrative format through character dialogue. To ensure narrative was an optimal message format for health information delivery, a nonnarrative cutscene of comparable character and background setting containing the same type of information about the health benefits of physical activity was produced as a control condition. In the narrative version, when stickmen started to attack the player, they explained the benefits of physical activity and encouraged the player to stay physically active. In the nonnarrative version, stickmen communicated to the player the same information. More details about the conditions can be found elsewhere [60].

Another 20 children from the Houston metro area were recruited to evaluate the narrative and nonnarrative cutscenes. Results indicated that children preferred the narrative cutscene over the nonnarrative cutscene [60]. The narrative version of *The Door* was burned onto a digital video disc (DVD) for use in the study.

### Implementation

A total of 40 children from the Houston metro area were recruited. Of these children, 8 participated in a pilot-testing session and 32 participated in the main study. The research protocol did not change between the pilot testing and main study. Thus, results were combined. Children were brought to the Metabolic Research Unit (MRU) of the Children’s Nutrition Research Center located in the Texas Medical Center in Houston, Texas. The MRU consisted of a private, semiopen space simulating a modern-day living room with home furniture and household electronics (eg, television) with a separate waiting room attached. Children played the AVG inside the room, while their parents stayed in the waiting room.

After consent and assent and facility orientation, children were randomly assigned to 1 of 2 conditions (narrative or nonnarrative) with 2 physical activity measurement instruments attached by a trained research associate. Those in the narrative condition first watched *The Door* (3 minutes in length) on a large LCD (liquid crystal display) television. The research associate then remotely switched the display from the DVD player to a Wii console preloaded with the *Swordplay: Showdown* AVG. The child was instructed to play the game for as long as he or she desired, up to a maximum playtime of 30 minutes. The research associate exited the room during AVG play to avoid social facilitation and observed the child play from a hidden monitor. The research protocol for those in the nonnarrative condition was identical, except that the children did not view the narrative cutscene before playing the AVG.

### Incentives

Each of the 40 children participating in the AVG play study session received a US $25 gift card.

### Measures

Children’s BMI was calculated as weight in kilograms divided by height in meters squared [61]. Children’s height and weight were measured twice by a research associate. A portable stadiometer (Shorr Height Measuring Board, Weigh and Measure, LLC, Olney, MD, USA) was used to measure height to the nearest 0.1 cm. Children’s weight was measured to the nearest 0.1 kg using a calibrated scale (Seca 813 digital floor scale, Seca GmbH & Co KG, Hamburg, Germany). The mean of the 2 recordings was recorded. A third measurement was taken in the event of a >0.2 cm or >0.2 kg difference between the first 2 measurements; the mean of these 3 measurements was taken. Each child’s age- and sex-specific BMI percentile was obtained from the Centers for Disease Control and Prevention manual [61].

Traditionally, activity sensors have been worn on the hip or arm areas for physical activity assessment. To measure physical activity during children’s AVG play, a Sensewear Pro Armband [62] (Sensewear, Jawbone, San Francisco, CA, USA) and ActiGraph GT3X+ triaxial accelerometer [63] (ActiGraph, ActiGraph, LLC, Pensacola, FL, USA) were attached to children (Sensewear Pro on their upper arms and ActiGraph on their hips). The devices were synchronized to ensure they recorded similar time intervals. The research associate documented the duration of the children’s AVG play time with a stopwatch. Step data were obtained from both the Sensewear Pro Armband and the ActiGraph accelerometer. Energy expenditure in joules was tracked through the Sensewear Pro Armband.

Social desirability of responses was assessed with the Revised Children’s Manifest Anxiety Scale (Sample item: I never say things I shouldn’t.) [64]. The questions were collected via a touch-screen tablet.

### Statistical Analysis

Independent *t* tests were performed to detect between-group differences in demographic information and BMI. For physical activity measurement, 2 phases were adopted for inferential statistical comparisons of the between-group difference using 2-sample *t* tests assuming unequal variances. Step 1 tested for significant differences in demographic (eg, age) and anthropometric (eg, BMI) measures to determine if the randomization process resulted in any biases in group physical characteristics. Step 2 compared play characteristics in terms of play duration, number of steps, and energy expenditure.

Power analysis showed that with 40 participants (20 per randomized sequence) and an alpha of .05, a 2-sided independent *t* test of the between-group narrative effect had 80% power to detect a large effect size of 0.91 SD units between means of the 2 conditions (narrative vs nonnarrative).

Independent 2-sample *t* tests and chi-square tests showed no between-group differences regarding demographic and baseline anthropometric measures of height, weight, BMI, BMI percentile, or social desirability. Because there was no group bias in participant height, weight, or demographics, covariance analysis was not conducted.

To test whether the narrative cutscene resulted in a more even distribution of activity over the play period, information entropy was used to analyze the probability distribution of the activity monitor data. Higher entropy indicates that the data are more evenly distributed, whereas lower entropy values indicate that activity was clustered around a narrow activity range.

## Results

The demographic information for the 40 children can be found in Table 1. The children were on average 9.6 years old and were evenly distributed on the basis of sex. They were from diverse backgrounds, with an overrepresentation of African and Hispanic American children (31/40, 78%). All children were overweight or obese (BMI = 27.2, BMI percentile = 94.3). Most parents (31/40, 78%) had attended some college or beyond. Children primarily resided in single-family homes (35/40, 88%) and lived with 2 or more adults in the household (34/40, 85%). When asked how they liked being in this project at the end of game play, all expressed that participating in the project was a positive experience and that they would like to participate in similar projects in the future.

**Table 1:** Children’s demographic and weight information (N=40).

Measure		Count, n	% or mean (SD)
**Sex (%)**			
	Female	20	50
	Male	20	50
**Race (%)**			
	Asian	2	5
	African American	14	35
	White American	6	15
	Hispanic American	17	43
	Multiracial	1	2
Age in years, mean (SD)		N/A^a^	9.6 (1.2)
BMI^b^ in kg/m^2^, mean (SD)		N/A	27.2 (11.9)
BMI percentile, mean (SD)		N/A	94.3 (12)
**Parent education (%)**			
	Eighth grade or less	1	2
	Some high school	1	2
	High school	4	10
	Technical school	3	8
	Some college	9	22
	College	11	28
	Postgraduate	11	28
**Annual income in US $ (%)**			
	< 20,000	5	12
	20,000-39,999	7	17
	40,000-59,999	12	30
	60,000-79,999	5	13
	80,000-100,000	5	13
	>100,000	6	15
**Type of residence (%)**			
	Single-family house	35	88
	Apartment	3	7
	Other	2	5
**Number of adults living in the household (%)**			
	1	6	15
	2	27	68
	3	7	17

^a^N/A: not applicable.

^b^BMI: Body mass index.

**Table 2:** Children’s physical activity levels during game play (N=40).

Device	Variable	Narrative (n=20), mean (SD)	Nonnarrative (n=20), mean (SD)	*t*_19_	*P* value
Stopwatch	Playing duration	17.6 (3.9)	16.2 (4.1)	1.14	.26
ActiGraph	Mean steps/10 seconds^a^	3.2 (0.7)	2.7 (0.7)	2.22	.03
	Total steps^a^	523.0 (203.3)	366.4 (172.0)	2.63	.01

^a^*P*<.05.

We found that the Sensewear Pro Armband significantly overestimated physical activity (ie, total energy expenditure was more than 500 kcal for a 20-minute AVG play) and that the step count correlations between the Sensewear and ActiGraph were inconsistent for the steps per 10 seconds (*r*=.39) and total steps (*r*=.32). Thus, data from Sensewear were discarded for further analysis.

ActiGraph more accurately assessed trunk movement. This is important because many children jumped around when playing with the Wii Remotes [65]. According to Table 2, when physical activity was measured with the ActiGraph accelerometer, children in the narrative group had significantly (*P*<.05) more steps during AVG play in terms of the average number of steps per 10-second period (mean 3.2, SD 0.7) and overall (mean 523, SD 203) when compared with the nonnarrative group (10-second period: mean 2.7, SD 0.7; overall: mean 366, SD 172).

Children in the narrative group had significantly lower entropy (mean 0.77, SD 0.14) than the nonnarrative group (mean 0.88, SD 0.15) according to the ActiGraph measurement (*P*=.01 measured by bits of information), that is, the narrative group’s physical activity data were clustered around fewer physical activity levels, indicating more consistent physical activity or play at a steadier pace. The standard deviation and coefficient of variation of the physical activity were not significantly different (*P*=.8 and *P*=.13, respectively). These results indicated that the participants’ activity patterns were not normally distributed. Thus the entropy analysis was warranted as a measure of variability.

Differences in average play duration (narrative: mean 17.6, SD 3.9 vs nonnarrative: mean 16.2, SD 4.1) and total energy expenditure (narrative: mean 566.9, SD 215.3 vs nonnarrative: mean 495.8, SD 190.7) were not statistically significant between the narrative and nonnarrative groups.

## Discussion

### Principal Findings

To the best of our knowledge, this is the first feasibility study of the influence of a narrative on physical activity during AVG play among overweight and obese children. Participants were from diverse backgrounds in an urban area and responded well to the project. The narrative was carefully developed with multiple empirical tests to ensure that plot and characters were closely related to the AVG and were positively received by the diverse child participants. Compared with the original nonnarrative AVG, the addition of a 3-minute narrative cutscene at the beginning of the game play session increased physical activity in children’s AVG play, as evidenced by increased average number of steps per 10 seconds and the total step counts. Our findings suggest that the participants in the narrative group were more physically active during game play and more effectively engaging their bodies in swordplay movements than those in the nonnarrative group, who played the original version of the AVG without a narrative cutscene. These findings provide preliminary evidence that an engaging narrative may influence child physical activity during AVG game play.

Children are imaginative beings who could be positively influenced by a make-believe world when a compelling narrative has been developed to meet their developmental, emotional, and recreational needs [66]. To respond to their needs, the research group conducted extensive formative work to ensure children were involved in narrative development, that character and plot design were child-friendly, and that the narrative was appealing. Thus, when a narrative cutscene corresponds to the original AVG and is engaging, children could be motivated to mentally incorporate the narrative into their active play. Future studies should investigate psychobehavioral mechanisms behind such an effect with the goal of maximizing narrative’s impact.

There are several limitations to this study. The scale of this study was small, with a small sample of children playing a single AVG session using multiple measurement devices attached to their body after watching just a 3-minute narrative cutscene in a research laboratory. Because of the feasibility nature of the study, the sample of 40 in this study was initially powered to detect a large difference; future studies should be powered to detect smaller differences. Measurement device placements and the laboratory setting may have caused participants to shorten their natural AVG play time, which could have reduced our ability to detect differences in energy expenditure and AVG play motivation between the conditions. Other studies have found similar unreliable measurement results by Sensewear Armbands among overweight and obese children [67,68], possibly because children tend to have a higher body temperature than adults and that changes in skin temperature are central to Sensewear Armbands’ energy expenditure calculation. These results suggested that measurement devices should be coordinated to accommodate specific physical activity measurement scenarios and should be able to accurately track physical activity among children. Having heard the story once, the attractiveness of the story may decrease for children who would like to play the game a second time. This may suggest branching narratives or randomized multiple plotlines. In addition, performance-based narrative development may motivate children to repeat AVG game play. Future studies should try measuring participants’ physical activity level in a more natural setting for elongated and multiple repeated study sessions and for increased frequency of each play session.

### Conclusions

This is the first feasibility study to systematically vary and test the effect of narrative on children’s physical activity during AVG play. Narrative increased physical activity during AVG game play among overweight and obese children as evidenced by more steps per 10-second period and total steps overall. Future research is needed to identify the underlying mechanisms through which this occurs.

## Abbreviations

AVG: active video game
BMI: body mass index
DVD: digital video disc
MRU: Metabolic Research Unit

## References

[1] Kaiser Family Foundation (2010). KFF.

[2] Vandewater EA, Shim M, Caplovitz AG (2004). Linking obesity and activity level with children's television and video game use. J Adolesc.

[3] Baranowski T, Baranowski J, Thompson D, Buday R, Jago R, Griffith MJ, Islam N, Nguyen N, Watson KB (2011). Video game play, child diet, and physical activity behavior change a randomized clinical trial. Am J Prev Med.

[4] Bailey BW, McInnis K (2011). Energy cost of exergaming: a comparison of the energy cost of 6 forms of exergaming. Arch Pediatr Adolesc Med.

[5] Biddiss E, Irwin J (2010). Active video games to promote physical activity in children and youth: a systematic review. Arch Pediatr Adolesc Med.

[6] Graf DL, Pratt LV, Hester CN, Short KR (2009). Playing active video games increases energy expenditure in children. Pediatrics.

[7] Foley L, Maddison R (2010). Use of active video games to increase physical activity in children: a (virtual) reality?. Pediatr Exerc Sci.

[8] Abernathy T, Rouse R (2014). GDC Vault.

[9] Jameson F (1981). The political unconscious: narrative as a socially symbolic act.

[10] Lu AS (2015). Narrative in Exergames: Thoughts on Procedure, Mechanism, and Others. Games Health J.

[11] Ogden CL, Carroll MD, Kit BK, Flegal KM (2014). Prevalence of childhood and adult obesity in the United States, 2011-2012. JAMA.

[12] Steinbeck KS (2001). The importance of physical activity in the prevention of overweight and obesity in childhood: a review and an opinion. Obes Rev.

[13] National Cancer Institute (2010). NCI.

[14] McTiernan A (2006). Cancer Prevention and Management through Exercise and Weight Control.

[15] Danaei G, Ding EL, Mozaffarian D, Taylor B, Rehm J, Murray CJ, Ezzati M (2009). The preventable causes of death in the United States: comparative risk assessment of dietary, lifestyle, and metabolic risk factors. PLoS Med.

[16] Freedman DS, Khan LK, Dietz WH, Srinivasan SR, Berenson GS (2001). Relationship of childhood obesity to coronary heart disease risk factors in adulthood: the Bogalusa Heart Study. Pediatrics.

[17] U.S. Department of Health and Human Services (2016). Health.gov.

[18] National Physical Activity Plan (2016). Physicalactivityplan.

[19] Shaya FT, Flores D, Gbarayor CM, Wang J (2008). School-based obesity interventions: a literature review. J Sch Health.

[20] Brown T, Summerbell C (2009). Systematic review of school-based interventions that focus on changing dietary intake and physical activity levels to prevent childhood obesity: an update to the obesity guidance produced by the National Institute for Health and Clinical Excellence. Obes Rev.

[21] Salmon J, Booth ML, Phongsavan P, Murphy N, Timperio A (2007). Promoting physical activity participation among children and adolescents. Epidemiol Rev.

[22] Biddiss E, Irwin J (2010). Active video games to promote physical activity in children and youth: a systematic review. Arch Pediatr Adolesc Med.

[23] (2014). Theesa.

[24] Greenwald W (2013). PC Magazine.

[25] Rideout V (2015). Commonsensemedia.

[26] Miles R (2008). Neighborhood disorder, perceived safety, and readiness to encourage use of local playgrounds. Am J Prev Med.

[27] Weir LA, Etelson D, Brand DA (2006). Parents' perceptions of neighborhood safety and children's physical activity. Prev Med.

[28] Maddison R, Foley L, Ni MC, Jiang Y, Jull A, Prapavessis H, Hohepa M, Rodgers A (2011). Effects of active video games on body composition: a randomized controlled trial. Am J Clin Nutr.

[29] O'Loughlin EK, Dugas EN, Sabiston CM, O'Loughlin JL (2012). Prevalence and correlates of exergaming in youth. Pediatrics.

[30] Fullerton S, Taylor AW, Dal GE, Berry N (2014). Measuring physical inactivity: do current measures provide an accurate view of “sedentary” video game time?. J Obes.

[31] DeMaria R, Wilson J (2002). High score! the illustrated history of electronic games.

[32] Chin A Paw MJ, Jacobs WM, Vaessen EP, Titze S, van Mechelen (2008). The motivation of children to play an active video game. J Sci Med Sport.

[33] Madsen KA, Yen S, Wlasiuk L, Newman TB, Lustig R (2007). Feasibility of a dance videogame to promote weight loss among overweight children and adolescents. Arch Pediatr Adolesc Med.

[34] Graves LE, Ridgers ND, Atkinson G, Stratton G (2010). The effect of active video gaming on children's physical activity, behavior preferences and body composition. Pediatr Exerc Sci.

[35] Kharrazi H, Lu AS, Gharghabi F, Coleman W (2012). A Scoping Review of Health Game Research: Past, Present, and Future. Games Health J.

[36] Lu AS, Kharrazi H, Gharghabi F, Thompson D (2013). A Systematic Review of Health Videogames on Childhood Obesity Prevention and Intervention. Games Health J.

[37] Fisher W (1985). The Narrative Paradigm: In the Beginning. J Communication.

[38] Rimmon-Kenan S (2002). Narrative Fiction: Contemporary Poetics.

[39] Cosmides L, Tooby J, Lewis M, Haviland-Jones JM (2000). Evolutionary psychologythe emotions. Handbook of Emotions.

[40] Kreuter MW, Green MC, Cappella JN, Slater MD, Wise ME, Storey D, Clark EM, O'Keefe DJ, Erwin DO, Holmes K, Hinyard LJ, Houston T, Woolley S (2007). Narrative communication in cancer prevention and control: a framework to guide research and application. Ann Behav Med.

[41] Green MC, Garst J, Brock TC, Shrum LJ (2004). The power of fiction: Determinants and boundaries. The Psychology of Entertainment Media: Blurring the Lines between Entertainment and Persuasion.

[42] Fazio R, Zanna M, Berkowitz L (1981). Direct experience and attitude-behavior consistency. Advances in Experimental Social Psychology.

[43] Epstein S, Barone DF, Hersen M, Van Hasselt VB (1998). Cognitive-experiential self-theory. Advanced personality.

[44] Oatley K, Green MC, Strange JJ, Brock TC (2002). Emotions and the story worlds of fiction. Narrative Impact: Social and Cognitive Foundations.

[45] Pillay H (2002). An investigation of cognitive processes engaged in by recreational computer game players: Implications for skills of the future. Journal of Research on Technology in Education.

[46] Slater M, Green MC, Strange JJ, Brock TC (2002). Entertainment education and the persuasive impact of narratives. Narrative Impact: Social and Cognitive Foundations.

[47] Cohen J (2001). Defining identification: A theoretical look at the identification of audiences with media characters. Mass Communication & Society.

[48] McLellan H (1993). Hypertextual Talestory Models for Hypertext Design. Journal of Educational Multimedia and Hypermedia.

[49] Laurillard D (1998). Multimedia and the learner's experience of narrative. Computers and Education.

[50] Bielenberg D, Carpenter-Smith T (1997). Efficacy of story in multimedia training. Journal of Network and Computer Applications.

[51] Whitaker RC, Wright JA, Pepe MS, Seidel KD, Dietz WH (1997). Predicting obesity in young adulthood from childhood and parental obesity. N Engl J Med.

[52] Gutin B, Islam S, Treiber F, Smith C, Manos T (1995). Fasting insulin concentration is related to cardiovascular reactivity to exercise in children. Pediatrics.

[53] McMurray RG, Bauman MJ, Harrell JS, Brown S, Bangdiwala SI (2000). Effects of improvement in aerobic power on resting insulin and glucose concentrations in children. Eur J Appl Physiol.

[54] Borgers N, de Leeuw E, Hox J (2000). Children as Respondents in Survey Research: Cognitive Development and Response Quality 1. Bulletin de Méthodologie Sociologique.

[55] (2011). CDC.

[56] Lu AS, Buday R, Thompson D, Baranowski T, Tettegah SY, Huang WD (2015). What type of narrative do children prefer in active video games? An exploratory study of cognitive and emotional responses. Emotions, Technology, and Digital Games.

[57] Lu AS (2009). What Race Do They Represent and Does Mine Have Anything to Do with It? Perceived Racial Categories of Anime Characters. Animation.

[58] Lu AS, Thompson D, Baranowski J, Buday R, Baranowski T (2012). Story Immersion in a Health Videogame for Childhood Obesity Prevention. Games Health J.

[59] Thompson D, Baranowski T, Buday R, Baranowski J, Thompson V, Jago R, Griffith MJ (2010). Serious Video Games for Health How Behavioral Science Guided the Development of a Serious Video Game. Simul Gaming.

[60] Fernandes DV, Mafra R, Beltran A, Baranowski T, Lu AS (2016). Children's Cognitive and Affective Responses About a Narrative Versus a Non-Narrative Cartoon Designed for an Active Videogame. Games Health J.

[61] (2011). CDC.

[62] (2010). Jawbone.

[63] ActiGraph (2011). Actigraphcorp.

[64] Reynolds C, Paget K (1983). National normative and reliability data for the Revised Children's Manifest Anxiety Scale. School Psychology Review.

[65] Ward DS, Evenson KR, Vaughn A, Rodgers AB, Troiano RP (2005). Accelerometer use in physical activity: best practices and research recommendations. Med Sci Sports Exerc.

[66] Nicolopoulou A, Bamberg M (1997). Children and narratives: Toward an interpretive and sociocultural approach. Narrative Development: Six Approaches.

[67] Dorminy CA, Choi L, Akohoue SA, Chen KY, Buchowski MS (2008). Validity of a multisensor armband in estimating 24-h energy expenditure in children. Med Sci Sports Exerc.

[68] Predieri B, Bruzzi P, Lami F, Vellani G, Malavolti M, Battistini NC, Iughetti L (2013). Accuracy of SenseWear Pro2 Armband to predict resting energy expenditure in childhood obesity. Obesity (Silver Spring).

